# Quality of Nutrition Environments, Menus and Foods Served, and Food Program Achievement in Oklahoma Family Child Care Homes

**DOI:** 10.3390/nu13124483

**Published:** 2021-12-15

**Authors:** Bethany D. Williams, Susan B. Sisson, Emily L. Stinner, Hope N. Hetrick, Marny Dunlap, Jennifer Graef-Downard, Kathrin Eliot, Karla Finnell, Alicia L. Salvatore

**Affiliations:** 1 Department of Nutritional Sciences, College of Allied Health, University of Oklahoma Health Sciences Center, 1200 N. Stonewall Ave., AHB 3068, Oklahoma City, OK 73117, USA; bethany.williams1@wsu.edu (B.D.W.); Emily.stinner@yahoo.com (E.L.S.); hope.hetrick@gmail.com (H.N.H.); Jennifer-GraefDownard@ouhsc.edu (J.G.-D.); Katie-Eliot@ouhsc.edu (K.E.); 2 Department of Nutrition and Exercise Physiology, Elson S. Floyd College of Medicine, Washington State University Health Sciences Spokane, 412 E Spokane Falls Blvd, Spokane, WA 99202, USA; 3 Department of Pediatrics, Section of General and Community Pediatrics, College of Medicine, University of Oklahoma Health Sciences Center, 1200 Children’s Ave., Oklahoma City, OK 73104, USA; Marny-Dunlap@ouhsc.edu; 4 Department of Health Promotion Sciences, Hudson College of Public Health, University of Oklahoma Health Sciences Center, 801 N.E. 13th Street, Oklahoma City, OK 73104, USA; Karla-Finnell@ouhsc.edu (K.F.); alicia.salvatore@christianacare.org (A.L.S.); 5 Institute for Research on Equity and Community Health, ChristianaCare, Wilmington, DE 19803, USA; 6 Department of Human Development and Family Sciences, University of Delaware, Newark, DE 19706, USA

**Keywords:** family child care homes, Child and Adult Care Food Program, nutrition environments, menu quality, dietary intake

## Abstract

Child care environments foster children’s healthy eating habits by providing exposure to healthy foods and feeding practices. We assessed the healthfulness of nutrition environments, menu/meal quality, and the achievement of Child and Adult Care Food Program (CACFP) guidelines and best practices in Oklahoma CACFP-enrolled family child care homes (FCCHs) (*n* = 51). Two-day classroom observations were conducted. Healthfulness of classroom nutrition environments was assessed using the Environment and Policy Assessment and Observation (EPAO). Foods served to and consumed by children were quantified using the Dietary Observations in Child Care (DOCC) tool. Nutrient analysis was performed to determine total energy for foods listed on menus, served to, and consumed by children. Menu and meal food variety and CACFP Guideline Achievement Scores were determined. Average nutrition environment score was 11.7 ± 1.2 (61.5% of maximum possible score). Energy (kcals) from menus and consumed by children was insufficient to meet two-thirds of their daily reference intake. Children were exposed to 1.7 vegetables and 1.3 fruits per meal. CACFP Guideline Achievement Scores were 66.3% ± 7.8 for menus and 59.3% ± 7.6 for mealtimes. Similar to previous research, our findings indicate a need for improved FCCH nutrition practices. Tailored interventions for FCCHs are needed.

## 1. Introduction

Early childhood is a critical period during which dietary patterns and eating habits are developed by young children [1]. Early care and education (ECE) settings, non-parental child care facilities, are critical environments that shape children’s nutrition and dietary behaviors. Approximately 60% of children spend time in ECE facilities; 33% of these children regularly attend for up to 35 h per week. Understanding ECE practices that are associated with child nutrition and are malleable to intervention is important for obesity prevention and overall health [2,3,4]. Improving the nutritional quality of foods served by ECE providers can promote a healthful lifestyle and shape positive dietary behaviors for children in care [5].

Various nutrition practices are recommended to enhance the quality of the overall ECE classroom “nutrition environment,” with ultimate goals to improve appropriate dietary intake, food preference, and lifelong health outcomes for young children. Specific practices to improve the nutrition environment include serving high-quality and a variety of foods [6,7,8]; providing healthy lifestyle learning materials in the classroom, such as nutrition-promoting books and posters [6]; and implementing staff nutrition behaviors [9]. Staff nutrition behaviors, also referred to as mealtime best practices, may include sitting with children during meals [9], enthusiastic role modeling of consuming healthful foods [10,11,12], involving children in meal preparation [13], and talking with children about healthy foods [14,15]. National organizations, including the Academy of Nutrition and Dietetics, American Public Health Association, the National Resource Center for Health and Safety in Child Care and Early Education, and the National Academy of Science (formerly known as the Institute of Medicine), strongly urge that ECE programs achieve recommended benchmarks for nutrition standards to assist public health efforts combatting childhood obesity [16,17,18]. In alignment with these goals, the United States Department of Agriculture (USDA) implemented the Child and Adult Care Food Program (CACFP) as a means to reimburse costs of higher-quality, nutritious foods for ECEs that primarily serve low-income children [19].

Barriers to improving nutrition environments and serving healthful foods vary by ECE context. Center-based child care settings function more like a school with classrooms, with more children overall and typically children of the same age [20]. In contrast, family child care homes (FCCHs) are often managed by a single non-relative provider who serves in multiple roles and cares for one or more children (6–12 maximum) of various ages in their own home [20,21]. Previous studies indicate differences in the quality of nutrition environments and in reported barriers to promoting ECE classroom health between centers and FCCHs. Statewide studies report that FCCH providers are more likely to serve fried, high-fat and/or high-sugar foods, and are more likely to report lack of time for preparing foods and sitting with children than are center-based child care providers [22,23,24]. Some evidence also suggests that children who attend FCCHs are more likely to become overweight or obese than are children attending child care centers [25]. Given these risks, and that FCCHs remain a relatively understudied ECE setting, there is a need to better understand current FCCH nutrition practices, particularly in states like Oklahoma that have high rates of childhood obesity [26]. Furthermore, since most previous research on FCCH nutrition environments has relied on self-reported data, there is a need for greater use of objective and observed measures [27]. 

Of the nearly 1500 FCCH providers in Oklahoma [28], 79% participate in CACFP [29]; however, there may be some discrepancies among the nutrition standards versus actual menus and foods served in FCCHs. While CACFP participation is associated with better self-reported adherence to nutrition standards in child care centers, there is room to improve implementation of practices to enhance the classroom nutrition environments overall, especially for staff nutrition behaviors that are recommended but do not affect reimbursement [24,30,31]. Few studies have described the quality of FCCH menus [32] or the quality of foods actually consumed by children in their care. It is vital to consider the quality of FCCH nutrition environments, menus, and foods served to and consumed by children to inform resources supporting child and public health, especially in FCCHs in Oklahoma, where residents are at high risk of obesity and related chronic outcomes [33]. This study addresses these gaps in the literature through two primary aims: (1) describe the healthfulness of Oklahoma FCCH overall nutrition environments, including foods/beverages served, staff nutrition behaviors, nutrition learning environment, and classroom healthy lifestyle materials; and (2) describe the quality of Oklahoma FCCH menus, foods served, and foods consumed by children, including nutrient composition, variety, and achievement of CACFP requirements and best practices.

## 2. Materials and Methods

This a cross-sectional analysis of baseline data collected in Happy Healthy Homes, a study of interventions to promote healthier FCCH environments [34]. To be eligible for this study, providers were required to: (1) be a licensed FCCH enrolled in the Child and Adult Care Food Program (CACFP); (2) provide care for at least one child between the ages of 2–5 years; (3) be located within a 60 mile radius of the University of Oklahoma Health Sciences Oklahoma City campus; and (4) prepare and serve meals for the children in their care. A total of 370 eligible FCCH providers were contacted through professional organization meetings, training events, emails, flyers, and direct calls made by the research team; 74 eligible providers consented to participate. Of these, 51 FCCH providers (68.9%) completed at least one day of baseline data collection, from which a total of 175 child dietary observations were conducted. All study procedures were approved by the University of Oklahoma Health Sciences Center Institutional Review Board (#7551). 

Trained observers collected baseline measures from October 2017 to October 2018. Visits were conducted on two non-consecutive unannounced dates within a one-week period. Observation visits began approximately one hour before the provider’s reported lunchtime and finished shortly after all children were done eating. The overall FCCH nutrition environment, including foods/beverages served and consumed, staff nutrition behaviors, nutrition learning environment, and healthy lifestyle materials displayed in the spaces of the home dedicated to learning (i.e., classroom), was assessed on both observation days. A single menu including at least 5 days of planned meals was collected. Types and quantities of foods served to and consumed by up to three 2-to-5-year-old children were recorded on both observation days.

### 2.1. Healthfulness of Nutrition Environments

Trained staff used the Environment and Policy Assessment and Observation (EPAO) to assess the FCCH nutrition environment during direct observation [6,35]. While the original full-day EPAO tool is comprised of 146 items, including nutrition and physical activity across a full day, a truncated version (30 items) was adapted for staff to observe the lunchtime and classroom nutrition environment specifically. The scoring tool consisted of seven subarea scores: (1) fruits and vegetables (range 0–20); (2) high-sugar/high-fat (range 0–20); (2) grains (range 0–20); (4) beverages (range 0–24); (5) staff nutrition behaviors (range 0–18); (6) nutrition learning environment (range 0–19); and (7) classroom healthy lifestyle materials (range 0–10). These seven subarea scores were averaged to calculate a total nutrition environment score (range 0–19), with a higher score indicating a more healthful FCCH nutrition environment. The validity and reliability of the full tool, and each subscale, are strong [35]. As presented in Vaughn et al., the intraclass correlations for the subscales used in our study range from 0.56 to 0.96, with individual item kappas ranging from 75.4 to 100.0 (with the majority in the 80s and 90s), and construct validity for the subscales was strong [35].

### 2.2. The Quality of Menus and Foods Served and Consumed

Menus were procured by researchers. Five days of a single menu, including breakfast, lunch, and afternoon snack, were analyzed for nutrient composition. Portion sizes for each food and meal were assumed as the CACFP required volume for 3-to-5-year-olds. Average nutrients provided per day were analyzed and combined for all three listed meals (breakfast, lunch and snack). Foods and beverages served and consumed during lunch was observed and documented using the Dietary Observations in Child Care (DOCC) [36] plate waste protocol. Unique identifiers were not collected on children; thus, the same or different children may have been observed on the two non-consecutive lunchtime observations. Food quantities were recorded through visual estimates in tablespoons, teaspoons, and quantity counts for a maximum of three 2-to-5-year-old children. Beverages were estimated visually in fluid ounces. Any foods and/or beverages that were dropped, spilled, traded, or taken from other children’s plates were observed and recorded. Observed plate waste has been validated [36] and is an accurate method to measure food consumption when compared with measured plate waste (*r* = 0.90–0.95) [37]. After confirming no difference in days and as per common procedure, mealtime nutrients were analyzed for each child, averaged per day, and days were averaged per site [38]. 

### 2.3. Nutrient Composition

Food Processor^TM^ Nutrition Analysis Software (ESHA, Salem, OR, USA) and the USDA food database were used to analyze the nutrient composition from 5-day menus and observed lunchtime foods. For nutrient analyses, a standard four-year-old female with a height of 40 inches and weighing 40 pounds was used as the referenced person. Product brands were included for food items, if possible; when specific brands or foods observed were not available in Food Processor^TM^, informed assumptions were made and tracked consistently across all providers. Total energy (kcals) was analyzed and compared with two-thirds and one-third children’s dietary reference intakes (DRIs for 1-to-3- and 4-to-8-year-old children) for menus and observed lunchtime foods, respectively, since the FCCH does not provide 100% of children’s nutrients [39]. 

### 2.4. Variety of Fruit and Vegetable Exposures

Fruit and vegetable variety was quantified by total number of exposures to different fruits and vegetables (canned, frozen, fresh, and dried) planned, served to, and consumed by children. For menus, each unique fruit and vegetable exposure was counted across all meals for five days. For foods served to and consumed by children, each unique fruit and vegetable exposure was counted across both observation days. Operationally, “fruit” included all canned, fresh, frozen, and dried fruits; “vegetables” included all dark green, red/orange, starchy, legumes, and other vegetables. Fried vegetables, small amounts of processed tomato products, pickles, and potato salad were not counted. 

### 2.5. CACFP Guidelines Achievement

CACFP Guideline Achievement Scores were determined using a measurement index assessing adherence to 2017 CACFP meal pattern requirements (i.e., type of fruits and vegetables served [fresh, frozen, canned, dried], type of meat/meat alternative served [lean, breaded, cheese, beans], juice, etc.) and best practices/recommendations (i.e., frequency of serving processed meat, percentage of fat in cheese, staff behaviors and feeding practices, seasonal and/or local produce, etc.) [40,41]. A score of 1 point was assigned for each item where the FCCH adhered to that specific CACFP guideline; thus, higher scores indicated higher CACFP achievement. All scores were analyzed as percentages of maximum points possible. Items to determine menu CACFP Requirements Scores (max. 26 points) were based on the menu for the same day as the first in-person observation visit. Items to determine menu CACFP Best Practice Scores (max. 25 points) were based on the full 5-day menu for the same week as the first in-person observation visit. Total Menu CACFP Achievement Score (max. 51 points) was the sum of both the Requirements and Best Practices Scores.

Items to determine meal CACFP Requirements Scores (max. 8 points), meal CACFP Best Practice Scores (max. 18 points), and Food Preparation Methods Scores (max. 2 points) were determined using lunch observations. These scores were summed to generate the total Mealtime CACFP Achievement Score (max. 28 points). Scores were calculated for each day, then averaged per site.

### 2.6. Statistical Analysis

The analytical sample included *n* = 51 providers who participated in at least one observation visit; *n* = 49 completed both observation visits and provided menus. To maximize the data set, two providers who did not complete the second observation were included with observation data analyzed based on a single day. For all other providers, EPAO scores and mealtime dietary data for each of two observation days were averaged and analyzed [42]. Descriptive characteristics, including means, medians, standard deviations, and frequencies, were calculated using SPSS 22.0 (IBM Corp, Armonk NY, USA) statistical analysis software. Nutrient analysis data were compared with two-thirds and one-third children’s dietary reference intakes (DRIs for 1-to-3- and 4-to-8-year-old children) for menus and observed lunchtime foods, respectively, using calculated 95% confidence intervals.

## 3. Results

Participating FCCH providers (*n* = 51) were aged 44.2 ± 14.2 years, mostly White (53.1%) or Black or African American (29.4%), and had completed at least some college or vocational training (63.3%) (Table 1). On average, FCCHs were in business for 10.8 ± 9.6 years, cared for an average number of 9.5 ± 4.2 children, and employed 1.4 ± 1.4 total staff.

**Figure 1:**
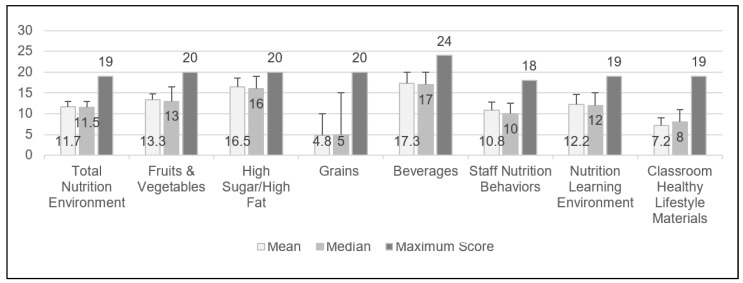
Nutrition environment total and sub scores of Oklahoma Family Child Care Homes as determined by the Environment and Policy Assessment and Observation (EPAO) tool (*n* = 51). (A second observation visit was not completed for two of the Family Child Care Home providers; therefore, the EPAO data during lunchtime were based on only one observation visit.)

The mean total nutrition environment score for participating FCCH programs averaged across two days was 11.7 ± 1.2 (61.5% of max. possible score; Figure 1). The highest nutrition subarea scores, relative to the respective possible maximum score, were the high-sugar/high-fat foods score (16.5 ± 2.0, 82.5% of max.) and healthful beverages score (17.3 ± 2.7, 72.0% of max.). The lowest nutrition subscores were for service of healthful grains (4.8 ± 5.2, 24.0% of max.) and classroom healthy lifestyle materials (7.2 ± 1.8, 37.8% of max.).

Energy (kcals) of foods planned on FCCH menus (including breakfast, lunch, and snack) was insufficient to meet two-third of the DRIs for both 1-to-3-year-old and 4-to-8-year-old children (Table 2). Energy (kcals) for mealtime foods served was within ranges to meet one-third of the DRIs for both child age ranges, suggesting that providers served more items than were indicated on menus (Table 3). However, energy (kcals) actually consumed by children at lunch was insufficient to meet DRIs. Across a 5-day menu, children were exposed to a higher variety of fruits (7.1 ± 1.8 per week) than vegetables (5.5 ± 2.1 per week; Table 4) throughout the week. When considering foods actually served at mealtimes, children were exposed to a higher variety of vegetables (1.7 per meal) than fruits (1.3 per meal). Children consumed fewer of the vegetables served (1.1 per meal, 62.2% consumed) than fruits served (1.1 per meal, 85.1% consumed).

The Total CACFP Guideline Achievement Scores were 66.3% ± 7.8 for menus and 59.3% ± 7.6 for menus and mealtimes (Table 5). While these scores were somewhat similar between menus and mealtimes, subscores varied. Menu subscores for CACFP requirements (65.8% ± 8.8) and best practices (66.7% ± 9.3) were relatively similar to each other and to the total achievement score. In contrast, mealtime scores varied across subcategories, including a much higher scores for CACFP requirements (82.7% ± 10.0) and lower scores for best practices (50.4% ± 8.9) and food preparation (46.1% ± 23.6).

## 4. Discussion

Childhood is a vital time to promote the development of healthy eating behaviors to improve children’s current and lifelong health. Nutrition environments where children spend a majority of their time, such as ECEs, have the potential to promote these health-enhancing behaviors [43]. The present study provides important insight into the nutritional health of FCCH classrooms, including the overall nutrition environment, the quality of foods planned, served, and consumed, and adherence to CACFP menu and meal guidelines. Our findings indicate several areas where FCCHs are successfully implementing nutrition practices to enhance the quality of the classroom nutrition environment. These include serving few high-sugar/high-fat foods, offering healthful beverages, providing sufficient energy at lunchtime, and adhering to mealtime CACFP requirements. However, our findings also reveal a number of areas for improvement. To improve the quality of FCCH nutrition environments, our findings suggest a need to improve the quality of grains offered to children, to offer healthy lifestyle materials in the classroom, to enhance the detail and the quality of planned menus, to increase the encouragement of children’s healthy eating behavior (i.e., adequate consumption of and variety of mealtime foods), and greater implementation of CACFP-recommended best practices. Our findings are consistent with previous literature reporting lower implementation of recommended nutrition practices in FCCHs than in other ECE contexts [44,45]. To date, there have been few nutrition education interventions offered to and evaluated specifically for FCCHs [34,46]. Our findings and those from previous research indicate a need for tailored interventions that will support FCCH providers in overcoming context-specific barriers for nutrition practices with lowest implementation.

In our study, FCCHs demonstrated the highest scores for implementing nutrition practices related to infrequent serving of high-sugar/high-fat foods and serving healthy beverages. Specific items contributing to these practice scores included limiting service of dessert or fried foods, preparing foods without fat or high-fat/-sugar condiments, increased availability of drinking water in the FCCH, and serving milk versus juice at lunchtime. This is not surprising, as these practices are directly related to CACFP requirements for foods offered to children. Furthermore, the CACFP does not provide reimbursement for grain-based desserts [47]. On average, the FCCH total nutrition environment score was 11.7 points out of the possible 19 (61.5%), indicating room for overall improvement. Our findings suggest, specifically, that health of the FCCH environments could be improved through providers serving whole grains more frequently throughout the day and providing children with healthy lifestyle materials, including books and posters promoting healthful foods and physical activity, in the classroom.

Additionally, our findings suggest discrepancies in the quality of foods listed on menus versus those actually served. Based on information provided on the 5-day menus, including breakfast, lunch, and snack, foods planned to be served to children were insufficient to meet energy (i.e., calorie needs) for both 1-to-3-year-old and 4-to-8-year-old children. However, this was not the case for foods actually served to children, which were within the range of meeting energy needs for both child age ranges. This finding may suggest that the CACFP minimum volume of food served, which was the assumed serving size for menu nutrient analyses, is insufficient to meet children’s energy needs. Further, while menus planned to serve a higher variety of fruits than vegetables, meals actually served to children contained a higher variety of vegetables than fruits. This may be due to higher service of fruits at breakfast and snacks increasing the variety of fruits, food which young children typically already like and consume in sufficient quantities [48]. FCCHs can be encouraged to serve more vegetables throughout the day, rather than predominantly at lunch. Finally, achievement of CACFP requirements was lower on average for menus than for foods served at mealtime, suggesting that, overall, food and beverages listed on menus were of lower quality than those that were actually served in the FCCH. These discrepancies are likely due to lack of specificity for listed menu items, as is permissible by non-specific CACFP menu guidelines. Previous research that has compared the quality of food planned on menus and food actually consumed has been mixed, with some studies indicating high agreement between foods on menus and those served [49], and some indicating discrepancies, likely due to a high frequency of CACFP-allowable substitutions during mealtimes [32,50]. While menus are important tools used to facilitate FCCH provider meal planning and communicate children’s in-care nutrition to parents, state regulations for menu reporting are inconsistent with national standards [51]. Further, menus lacking specificity and accuracy may negatively impact the accuracy of evaluation efforts for nutrition intervention in child care. If menus are to be used by parents, health professionals, and researchers for nutritional information, greater detail and specificity in menus is needed, although menus should still allow for the inclusion of seasonal and locally available foods.

Our findings suggest that the quality of foods and beverages, including variety of fruits and vegetables and total energy, were more desirable for meals planned on menus and served at lunchtime than for those actually consumed by children. Children did not consume the full variety of foods served, including vegetables (1.1 per meal, 62.2% of variety consumed) and fruits (1.1 per meal, 85.1% of variety consumed). Further, children did not consume sufficient energy to meet one-third of the DRIs for 1-to-3-year-old and 4-to-8-year-old children, even though sufficient energy was served at lunchtime. These results are consistent with previous studies noting that children may not consistently consume healthful foods served in child care [52]. Our findings additionally align with child care providers’ reporting that children’s refusal to taste new foods, or “picky eating,” is a significant barrier to serving healthful food items [22,53]. While children exposed to a higher variety of foods are more likely to consume a higher variety of food items overall [7,8], many FCCH providers are not aware that children need a minimum of 8–15 exposures to a food before acceptance [54]. Thus, FCCH providers should be encouraged to continue serving high-quality foods to children, while implementing healthful practices during mealtime to encourage children’s consumption.

Staff nutrition behaviors at mealtime are effective means to encourage children to try healthful, and often less preferred, food items [55]. FCCH provider feeding practices shape child eating behaviors through children’s learned preferences, and therefore may encourage consumption of the healthful foods they are served [5]. Implementation of these staff nutrition behaviors are associated with higher diet quality and healthier weight outcomes in children [14,56]. Conversely, when staff utilize more authoritarian or pressuring feeding tactics during mealtime (i.e., requiring children to clean their plates or take bites of foods after refusal) rather than promoting children’s autonomy, children in pressuring environments taste, on average, a lower variety of foods [15,56]. Consistent with previous literature [24], implementation of staff nutrition behaviors within our study FCCHs was also relatively low (10.8 points out of the possible 18, 60%). Specific staff behaviors for improvement included role modeling consumption of healthful foods, sitting with children during meals, cueing to children’s hunger and satiety, and using non-food items to reward good behavior [5,27]. Such improvements in staff nutrition behaviors have the potential to improve children’s intake of healthful foods served in child care and alleviate common barriers to providers serving those foods.

In our study, FCCH providers had lower achievement of CACFP requirements on menus (65.8% of maximum) compared with a higher achievement for mealtime (82.7% of maximum). Conversely, for CACFP best practices, a higher achievement was observed for menus (66.7% of maximum) than for mealtimes (50.4% of maximum). Adherence to CACFP requirements is related to the improved nutritional quality of meals served and consumed by young children in care [39,57,58]. These results add to the current literature while suggesting room for improvement in achieving CACFP guidelines. Similarly, previous studies have also reported higher accomplishment of CACFP requirements compared with best practices (i.e., food variety and staff nutrition behaviors) [24,32,41,52,59,60]. This is logical, given that reimbursement for food items is dependent on adherence to requirements, and not best practices. Finally, in our study, the CACFP subscore with lowest achievement was observed for mealtime healthful food preparation methods (46.1% of maximum); specifically, low scores were observed for those preparing foods with added fats or using heat and serve foods (i.e., prefried, frozen). To better align with CACFP guidelines, and thus best address inherent concerns for children’s health targeted by the federal food program, future promotion, and research studies in FCCHs should target improved food variety for meals and menus, use of staff nutrition behaviors, and healthfulness of meal preparation techniques.

## 5. Strengths and Limitations

A primary strength of our study is the focus and recruitment of FCCHs, a population underrepresented in the scientific literature. Research personnel were rigorously trained and certified in measurement procedures, which enhances data quality. Objective measures were conducted over two observation days and averaged to account for variability. One limitation is the lack of data collected to account for FCCHs’ differing child dynamics at each mealtime, including number of children and children’s age and sex. Menus did not necessarily reflect planned meals for the precise days of observation, which precluded comparison between what was planned and what was actually served and consumed. Menus also lacked specificity on types of food and beverages planned to be served; thus, we relied on consistent processing assumptions. Further, means of describing menu and meal quality are inconsistent across the literature. Therefore, the current study’s definition of quality may not be directly comparable to similar studies. Since our sample of FCCH providers was limited geographically (Oklahoma City metropolitan area) and all participated in the CACFP, this study may have limited generalizability. However, ongoing studies using the same measures, to be published separately, will assess nutritional environments of FCCHs across the state of Oklahoma, with efforts focused in rural areas.

## 6. Conclusions and Future Directions

This study described the overall nutrition environment, the quality of menus and foods served, and CACFP achievement in 51 FCCHs across the Oklahoma City Metropolitan area. Consistent with previous studies, our findings indicate room to improve the healthfulness of FCCH nutrition environments. Energy (kcals) from menus and consumed by children was insufficient to meet two-third of the DRIs for both 1-to-3-year-old and 4-to-8-year-old children, while energy served to children at mealtime was sufficient for both age groups. Children were exposed to a higher variety of vegetables (1.7 per meal) than fruits (1.3 per meal) during mealtime, though they were less likely to consume the full variety of vegetables served than to consume the full variety of fruits served. Total CACFP Achievement Scores were 66.3% ± 7.8 and 59.3% ± 7.6 for menus and mealtimes, respectively. Future intervention and policy should be tailored to the unique context of FCCHs. There are few studies that have examined together the foods and beverages served and consumed [5,15], and few comprehensive studies on nutrition environments focused on FCCHs. To capture a fuller picture of FCCH environments and experiences, future studies should include FCCHs in rural communities, those not participating in the CACFP, and unlicensed homes.

## Figures and Tables

**Table 1:** Demographic Characteristics of Family Child Care Home (FCCH) Providers in and around Oklahoma City, Oklahoma Participating in Happy Healthy Homes Baseline Measures Fall 2017–Fall 2018 (*n* = 51).

	Frequency or Mean ± SD	% or Range
**Provider**		
Age in years ^1^	44.2 ± 14.2	23–71
Race/Ethnicity		
Black or African American	15	29.4%
American Indian or Alaska Native	2	3.9%
White or Caucasian	26	50.9%
Asian, Pacific Islander	4	7.8%
Other, or did not wish to provide	6	11.7%
Missing	2	3.9%
Level of education		
High school graduate or GED	4	7.8%
Some college or vocational training	31	60.7%
4 year college graduate or higher	14	27.4%
Missing	2	3.9%
Household income		
≤$49,999	17	33.3%
$50,000 to $99,999	17	33.3%
≥$100,000	10	19.6%
Do not wish to provide	5	9.8%
Missing	2	3.9%
**Program**		
Years in business as a FCCH ^2^	10.8 ± 9.6	0.3–40
Number of children attending the FCCH ^2^	9.5 ± 4.2	3–24
Number of children ≤5 years old ^3^	6.8 ± 3.6	0–16
Number of staff ^2^	1.4 ± 1.4	0–5
Average hours preparing meals daily ^2^	1.9 ± 0.9	0.5–5

^1^ Seven missing FCCH providers (*n* = 44); ^2^ two missing FCCH providers (*n* = 49); ^3^ three missing FCCH providers (*n* = 48).

**Table 2:** Energy (kcals) of daily meals planned on menus of Oklahoma Family Child Care Homes (*n* = 49).

Nutrient	DRIs		Menu-Meals Planned				
	**1-to-3-Year-Old**	**4-to-8-Year-Old**	**Mean ± SD**	**Median ± IQR**	**95% CI**	**Within 95% CI for 1-to-3-Year-Old DRIs?**	**Within 95% CI for 4-to-8-Year-Old DRIs?**
Energy (kcals)	666–933.3	800–1066.7	624.17 ± 62.3	608.82 ± 78.2	606.2–642.0	Insufficient	Insufficient

DRIs, dietary reference intakes representing two-thirds of the daily recommended intake value. Kcals, kilocalories. SD, standard deviation. IQR, interquartile range. CI, confidence interval.

**Table 3:** Energy (kcals) of daily foods served and consumed in Oklahoma Family Child Care Homes (*n* = 51 ^a^).

Nutrient	DRIs		Mealtime-Foods Served					Mealtime-Foods Consumed				
	**1-to-3-Year-Old**	**4-to-8-Year-Old**	**Mean ± SD**	**Median ± IQR**	**95% CI**	**Within 95% CI for 1-to-3-Year-Old DRIs?**	**Within 95% CI for 4-to-8-Year-Old DRIs?**	**Mean ± SD**	**Median ± IQR**	**95% CI**	**Within 95% CI for 1-to-3-Year-Old DRIs?**	**Within 95% CI for 4-to-8-Year-Old DRIs?**
Energy (kcals)	333–466.7	400–533.4	387.0 ± 137.8	370.8 ± 176.6	349.1–424.8	Within	Within	269.1 ± 124.8	261.2 ± 159.9	234.8–303.3	Insufficient	Insufficient

DRIs, dietary reference intakes representing one-third of the daily recommended intake value. Kcals, kilocalories. SD, standard deviation. IQR, interquartile range. CI, confidence interval. ^a^ A second observation visit was not completed for two of the FCCH providers; therefore, mealtime foods served and consumed were based on only one observation visit.

**Table 4:** Number of exposures to types of fruits and vegetables planned on menus and served and consumed during mealtime in Oklahoma Family Child Care Homes (*n* = 51).

Food Subgroup	Menu-Meals Planned ^a^		Mealtime-Foods Served ^b^		Mealtime-Foods Consumed ^b^	
	**Mean ± SD**	**Median ± IQR**	**Mean ± SD**	**Median ± IQR**	**Mean ± SD**	**Median ± IQR**
Fruits	7.1 ± 1.8	7.0 ± 2.0	1.3 ± 0.6	1.0 ± 0.5	1.1 ± 0.5	1.0 ± 0.5
Vegetables	5.5 ± 2.1	6.0 ± 3.0	1.7 ± 1.2	1.5 ± 1.0	1.1 ± 0.7	1.0 ± 1.0

^a^*n* = 49. ^b^ A second observation visit was not completed for two of the Family Child Care Home providers; therefore, mealtime foods served and consumed were based on only one observation visit. SD, standard deviation. IQR, interquartile range.

**Table 5:** Mean Child and Adult Food Program (CACFP) achievement for requirements and recommended practices for menus and foods served and consumed during mealtime in Oklahoma Family Child Care Homes (*n* = 51).

CACFP Achievement Score	Menu-Meals Planned ^a^				Mealtime-Foods Served and Consumed ^b^			
	**Mean Score ± SD**	**Median Score ± IQR**	**Mean Percent ± SD**	**Median Percent ± IQR**	**Mean Score ± SD**	**Median Score ± IQR**	**Mean Percent ± SD**	**Median Percent ± IQR**
Total Score	33.8 ± 3.9	34.0 ± 6.0	66.3 ± 7.8	66.6 ± 10.7	16.6 ± 2.1	17.0 ± 3.0	59.3 ± 7.6	60.7 ± 10.7
Requirements	17.1 ± 2.2	17.0 ± 3.0	65.8 ± 8.8	65.3 ± 11.5	6.6 ± 0.8	7.0 ± 1.0	82.7 ± 10.0	87.5 ± 12.5
Best Practices	16.6 ± 2.3	16.0 ± 4.0	66.7 ± 9.3	64.0 ± 14.0	9.1 ± 1.6	9.0 ± 2.0	50.4 ± 8.9	50.0 ± 11.1
Food Preparation	n/a	n/a	n/a	n/a	0.9 ± 0.5	1.0 ± 0.5	46.1 ± 23.6	50.0 ± 25.0

^a^*n* = 49. ^b^ A second observation visit was not completed for two of the Family Child Care Home providers; therefore, mealtime foods served and consumed were based on only one observation visit. SD, standard deviation. IQR, interquartile range.

## Data Availability

The data presented in this study are available on request from the corresponding author. The data are not publicly available due to privacy protection for study participants.

## References

[1] Tovar A., Risica P., Mena N., Lawson E., Ankoma A., Gans K.M. (2015). An Assessment of Nutrition Practices and Attitudes in Family Child-Care Homes: Implications for Policy Implementation. Prev. Chronic Dis..

[2] Trost S.G., Messner L., Fitzgerald K., Roths B. (2009). Nutrition and Physical Activity Policies and Practices in Family Child Care Homes. Am. J. Prev. Med..

[3] Laughlin L.L. (2010). Who’s Minding the Kids? Child Care Arrangements: Spring 2005/Summer 2006.

[4] Mary P.D.S., Kaphingst K.M., French S. (2006). The Role of Child Care Settings in Obesity Prevention. Future Child..

[5] Tovar A., Benjamin-Neelon S.E., Vaughn A.E., Tsai M., Burney R., Østbye T., Ward D.S. (2018). Nutritional Quality of Meals and Snacks Served and Consumed in Family Child Care. J. Acad. Nutr. Diet..

[6] Tovar A., Vaughn A.E., Fisher J.O., Neelon S.E.B., Burney R., Webster K., Liu T., Ostbye T., Ward D.S. (2019). Modifying the Environment and Policy Assessment and Observation (EPAO) to better capture feeding practices of family childcare home providers. Public Health Nutr..

[7] Birch L.L. (1999). Development of food preferences. Annu. Rev. Nutr..

[8] Skinner J.D., Carruth B.R., Bounds W., Ziegler P.J. (2002). Children’s food preferences: A longitudinal analysis. J. Am. Diet. Assoc..

[9] Kharofa R.Y., Kalkwarf H.J., Khoury J.C., Copeland K.A. (2016). Are Mealtime Best Practice Guidelines for Child Care Centers Associated with Energy, Vegetable, and Fruit Intake?. Child. Obes..

[10] Hendy H.M. (1999). Comparison of five teacher actions to encourage children’s new food acceptance. Ann. Behav. Med..

[11] Hendy H., Raudenbush B. (2000). Effectiveness of teacher modeling to encourage food acceptance in preschool children. Appetite.

[12] Ward S.A., Bélanger M., Donovan D., Vatanparast H., Muhajarine N., Engler-Stringer R., Leis A., Humbert M.L., Carrier N. (2017). Association between childcare educators’ practices and preschoolers’ physical activity and dietary intake: A cross-sectional analysis. BMJ Open.

[13] Gubbels J.S., Gerards S.M., Kremers S.P. (2015). Use of Food Practices by Childcare Staff and the Association with Dietary Intake of Children at Childcare. Nutrients.

[14] Gubbels J.S., Kremers S.P.J., Stafleu A., Dagnelie P.C., De Vries N.K., Thijs C. (2010). Child-care environment and dietary intake of 2- and 3-year-old children. J. Hum. Nutr. Diet..

[15] Anundson K., Sisson S.B., Anderson M., Horm D., Soto J., Hoffman L. (2018). Staff Food-Related Behaviors and Children’s Tastes of Food Groups during Lunch at Child Care in Oklahoma. J. Acad. Nutr. Diet..

[16] Benjamin-Neelon S.E. (2018). Position of the Academy of Nutrition and Dietetics: Benchmarks for Nutrition in Child Care. J. Acad. Nutr. Diet..

[17] Burns A., Parker L., Birch L.L. (2011). Early Childhood Obesity Prevention Policies.

[18] Pediatrics AAO, Association APH (2021). Preventing Childhood Obesity in Early Care and Education Programs.

[19] Child and Adult Care Food Program (CACFP) (2018). Food and Nutrition Service.

[20] Natale R., Page M., Sanders L. (2014). Nutrition and Physical Activity Practices in Childcare Centers Versus Family Childcare Homes. J. Fam. Econ. Issues.

[21] Ogden C.L., Carroll M.D., Curtin L.R., Lamb M.M., Flegal K.M. (2010). Prevalence of High Body Mass Index in US Children and Adolescents, 2007–2008. JAMA.

[22] Dev D.A., Garcia A.S., Dzewaltowski D., Sisson S., Franzen-Castle L., Rida Z., Williams N.A., Hillburn C., Dinkel D., Srivastava D. (2020). Provider reported implementation of nutrition-related practices in childcare centers and family childcare homes in rural and urban Nebraska. Prev. Med. Rep..

[23] Williams B., Sisson S., Lowery B., Dev D., Horm D., Campbell J., Finneran D., Graef-Downard J. (2021). Associations Between Community Nutrition Environments and Early Care and Education Classroom Nutrition Practices. Curr. Dev. Nutr..

[24] Williams B.D., Sisson S.B., Padasas I.O., Dev D.A. (2021). Food Program Participation Influences Nutrition Practices in Early Care and Education Settings. J. Nutr. Educ. Behav..

[25] Swyden K., Sisson S.B., Castle S., Lora K.R., Copeland K. (2017). Child Care Status and Childhood Obesity: A Narrative Review of Literature. Int. J. Obes..

[26] Weedn A.E., Hale J.J., Thompson D.M., Darden P.M. (2014). Trends in Obesity Prevalence and Disparities among Low-Income Children in Oklahoma, 2005–2010. Child. Obes..

[27] Benjamin-Neelon S.E., Vaughn A.E., Tovar A., Østbye T., Mazzucca S., Ward D.S. (2018). The family child care home environment and children’s diet quality. Appetite.

[28] Faulkner L.A. (2020). Child Care Home and Capacity and Children in Subsidized Care as of December 2015. Personal Communication with Sisson SB.

[29] Weber J. (2020). Tier of FCCH Provider Reimbursement. Personal Communication with Sisson SB.

[30] Andreyeva T., Kenney E.L., O’Connell M., Sun X., Henderson K.E. (2018). Predictors of Nutrition Quality in Early Child Education Settings in Connecticut. J. Nutr. Educ. Behav..

[31] Erinosho T., Hales D., Vaughn A., Gizlice Z., Ward D. (2019). The Quality of Nutrition and Physical Activity Environments of Family Child-Care Homes in a State in the Southern United States. J. Acad. Nutr. Diet..

[32] Dave J.M., Cullen K.W. (2018). Foods Served in Child Care Facilities Participating in the Child and Adult Care Food Program: Menu Match and Agreement with the New Meal Patterns and Best Practices. J. Nutr. Educ. Behav..

[33] Whelan L., Hartwell M., Bell S.B., Thomas V., Wetherill M. (2019). Lifestyle Risk Factors and Chronic Disease in Oklahoma: A secondary analysis of the Behavioral Risk Factor Surveillance Survey 2017. J. Okla. State Med. Assoc..

[34] Sisson S., Salvatore A.L., Hildebrand D., Poe T., Merchant C., Slawinski M., Kracht C.L., Stoner J.A., Lazarte N.A., Schneider L.A.F. (2019). Interventions to promote healthy environments in family child care homes in Oklahoma—Happy Healthy Homes: Study protocol for a randomized controlled trial. Trials.

[35] Vaughn A.E., Mazzucca S., Burney R., Østbye T., Neelon S.E.B., Tovar A., Ward D.S. (2017). Assessment of nutrition and physical activity environments in family child care homes: Modification and psychometric testing of the Environment and Policy Assessment and Observation. BMC Public Health.

[36] Ball S.C., Benjamin S.E., Ward D.S. (2007). Development and Reliability of an Observation Method to Assess Food Intake of Young Children in Child Care. J. Am. Diet. Assoc..

[37] Comstock E.M., Pierre R.G.S., Mackiernan Y.D. (1981). Measuring individual plate waste in school lunches. Visual estimation and children’s ratings vs. actual weighing of plate waste. J. Am. Diet. Assoc..

[38] Rasbold A.H., Adamiec R., Anderson M.P., Campbell E.J., Horm D.M., Sitton L.K., Sisson S.B. (2016). Macronutrient and micronutrient intakes of children in Oklahoma child-care centres, USA. Public Health Nutr..

[39] Maalouf J., Evers S.C., Griffin M., Lyn R. (2013). Assessment of Mealtime Environments and Nutrition Practices in Child Care Centers in Georgia. Child. Obes..

[40] Sisson S., Sleet K., Rickman R., Love C., Williams M., Jernigan V.B.B. (2019). The development of child and adult care food program best-practice menu and training for Native American head start programs: The FRESH study. Prev. Med. Rep..

[41] Sisson S.B., Sleet K., Rickman R., Love C., Bledsoe A., Williams M., Jernigan V.B.B. (2019). Impact of the 2017 Child and Adult Care Food Program Meal Pattern Requirement Change on Menu Quality in Tribal Early Care Environments: The Food Resource Equity and Sustainability for Health Study. Curr. Dev. Nutr..

[42] Fleischhacker S., Cason K.L., Achterberg C. (2006). “You Had Peas Today?”: A Pilot Study Comparing a Head Start Child-Care Center’s Menu with the Actual Food Served. J. Am. Diet. Assoc..

[43] Kumar S., Kelly A.S. (2017). Review of Childhood Obesity: From Epidemiology, Etiology, and Comorbidities to Clinical Assessment and Treatment. Mayo Clin. Proc..

[44] Gerritsen S., Dean B., Morton S.M., Wall C. (2017). Do childcare menus meet nutrition guidelines? Quantity, variety and quality of food provided in New Zealand Early Childhood Education services. Aust. N. Z. J. Public Health.

[45] Martyniuk O.J., Vanderloo L.M., Irwin J.D., Burke S.M., Tucker P. (2016). Comparing the nutrition environment and practices of home- and centre-based child-care facilities. Public Health Nutr..

[46] Risica P.M., Tovar A., Palomo V., Dionne L., Mena N., Magid K., Ward D.S., Gans K.M. (2019). Improving nutrition and physical activity environments of family child care homes: The rationale, design and study protocol of the ‘Healthy Start/Comienzos Sanos’ cluster randomized trial. BMC Public Health.

[47] U.S. Department of Agriculture, Center for Nutrition Policy and Promotion (2010). Development of 2010 Dietary Guidelines for Americans Consumer Messages and New Food Icon.

[48] Kim S.A., Moore L.V., Galuska D., Wright A.P., Harris D., Grummer-Strawn L.M., Merlo C.L., Nihiser A.J., Rhodes D.G. (2014). Division of Nutrition, Physical Activity, and Obesity, National Center for Chronic Disease Prevention and Health Promotion, CDC. Vital signs: Fruit and vegetable intake among children—United States, 2003–2010. MMWR Morb. Mortal Wkly. Rep..

[49] Breck A., Dixon L.B., Khan L.K. (2016). Comparison of planned menus and centre characteristics with foods and beverages served in New York City child-care centres. Public Health Nutr..

[50] Benjamin Neelon S.E.C.K., Ball S.C., Bradley L., Ward D.S. (2010). Comparison of Menus to Actual Foods and Beverages Served in North Carolina Child-Care Centers. J. Am. Diet. Assoc..

[51] Benjamin S.E., Copeland K.A., Cradock A., Neelon B., Walker E., Slining M.M., Gillman M.W. (2009). Menus in Child Care: A Comparison of State Regulations with National Standards. J. Am. Diet. Assoc..

[52] Erinosho T., Dixon L.B., Young C., Brotman L.M., Hayman L.L. (2011). Nutrition Practices and Children’s Dietary Intakes at 40 Child-Care Centers in New York City. J. Am. Diet. Assoc..

[53] Zaltz D.A., Pate R.R., O’Neill J.R., Neelon B., Benjamin-Neelon S.E. (2018). Barriers and Facilitators to Compliance with a State Healthy Eating Policy in Early Care and Education Centers. Child. Obes..

[54] Carruth B.R., Ziegler P.J., Gordon A., Barr I.S. (2004). Prevalence of picky eaters among infants and toddlers and their caregivers’ decisions about offering a new food. J. Am. Diet. Assoc..

[55] Dovey T., Staples P.A., Gibson E.L., Halford C.G. (2008). Food neophobia and ’picky/fussy’ eating in children: A review. Appetite.

[56] Mennella J.A., Ventura A.K. (2011). Early Feeding: Setting the Stage for Healthy Eating Habits. Limits Hum. Endur..

[57] Bruening K.S., Gilbride J.A., Passannante M.R., McCLOWRY S. (1999). Dietary Intake and Health Outcomes among Young Children Attending 2 Urban Day-care Centers. J. Am. Diet. Assoc..

[58] Oakley C.B., Bomba A.K., Knight K.B., Byrd S.H. (1995). Evaluation of Menus Planned in Mississippi Child-Care Centers Participating in the Child and Adult Care Food Program. J. Am. Diet. Assoc..

[59] Monsivais P., Kirkpatrick S., Johnson D.B. (2011). More Nutritious Food Is Served in Child-Care Homes Receiving Higher Federal Food Subsidies. J. Am. Diet. Assoc..

[60] Schwartz M.B., Henderson K.E., Grode G., Hyary M., Kenney E.L., O’Connell M., Middleton A.E. (2015). Comparing Current Practice to Recommendations for the Child and Adult Care Food Program. Child. Obes..

